# Robust Bioinspired MXene–Hemicellulose Composite Films with Excellent Electrical Conductivity for Multifunctional Electrode Applications

**DOI:** 10.1021/acsnano.2c08163

**Published:** 2022-10-26

**Authors:** Ruwei Chen, Hao Tang, Yuhang Dai, Wei Zong, Wei Zhang, Guanjie He, Xiaohui Wang

**Affiliations:** † State Key Laboratory of Pulp and Paper Engineering, South China University of Technology, Guangzhou 510640, China; ‡ Christopher Ingold Laboratory, Department of Chemistry, University College London, 20 Gordon Street, London WC1H 0AJ, U.K.; § Electrochemical Innovation Lab, Department of Chemical Engineering, University College London, London WC1E 7JE, U.K.; ∥ School of Engineering and Materials Science, Queen Mary University of London, London E1 4NS, United Kingdom

**Keywords:** MXene, hemicellulose, structural electrode, mechanical strength, electrical conductivity, supercapacitor, humidity sensor

## Abstract

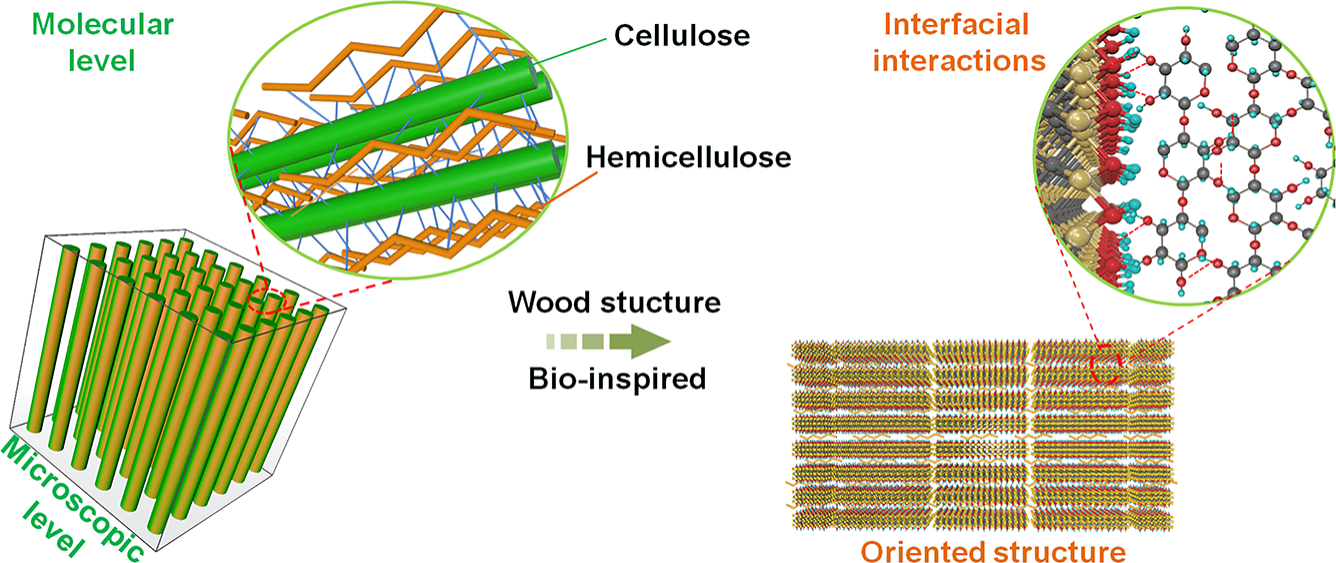

MXene-based structural materials with high mechanical robustness and excellent electrical conductivity are highly desirable for multifunctional applications. The incorporation of macromolecular polymers has been verified to be beneficial to alleviate the mechanical brittleness of pristine MXene films. However, the intercalation of a large amount of insulating macromolecules inevitably compromises their electrical conductivity. Inspired by wood, short-chained hemicellulose (xylo-oligosaccharide) acts as a molecular binder to bind adjacent MXene nanosheets together; this work shows that this can significantly enhance the mechanical properties without introducing a large number of insulating phases. As a result, MXene–hemicellulose films can integrate a high electrical conductivity (64,300 S m^–1^) and a high mechanical strength (125 MPa) simultaneously, making them capable of being high-performance electrode materials for supercapacitors and humidity sensors. This work proposes an alternative method to manufacture robust MXene-based structural materials for multifunctional applications.

Emerging flexible electronics have greatly stimulated the demand for structural electrode materials with integrated high electrical conductivity and mechanical strength for multifunctional applications.^1^ Ti_3_C_2_T_*x*_, the first member of the MXenes with metallic conductivity and mixed surface terminations, has stimulated a wide range of innovations, such as electrochemical energy storage, optoelectronics, and electromagnetic interference shielding.^2−6^ However, the fragile nature of pristine MXene films, due to the poor interaction between adjacent nanosheets, restricts their applications in structural electrode materials.

The incorporation of polymers, including sodium alginate, poly(vinyl alcohol), cellulose nanofibers, and aramid nanofibers, can regulate the mechanical performance of MXene films.^7−12^ Although improved mechanical performance could be achieved, the intercalation of a large amount of insulating macromolecules inevitably compromises the electrical conductivities by several orders of magnitude. In addition, hybridizing MXene with other conductive nanomaterials such as carbon nanotubes, graphene, polypyrrole, polyaniline, and Ag nanowires could only increase the electrochemical performance or alleviate the deterioration of electrical conductivity while slightly contributing to the improvement of mechanical properties.^13−20^ Therefore, improving mechanical properties while maintaining high electrical conductivity has been a big challenge for MXene film modification.

In nature, the evolutionary selection pressure has promoted natural materials to optimize their structures from molecular to macroscopic levels to adapt to environmental conditions.^21^ For instance, as an abundant natural resource, wood has been widely utilized as an engineering material due to its sophisticated hierarchical structures with high strength and toughness.^22^ At the microscopic level, the cell walls of wood consist of oriented semicrystalline cellulose fibrils encapsulated in an amorphous lignin–hemicellulose matrix (Figure 1a), which make the wood a “reinforced concrete” structure, strengthening and stiffening the wood against environmental stress.^23^ At the molecular scale, hemicellulose with abundant side groups and segment polarity can serve as a compatibilizer at the lignin and the cellulose interface through the hydrogen bond and dipole–dipole interactions, thereby improving the total cohesiveness and compatibility.^24^ Moreover, another part of the hemicellulose is embedded within cellulose domains, bonding with adjacent cellulose fibril aggregates through van der Waals and hydrogen bondings (Figure 1a).^25^ Therefore, these oriented structures at the microscopic level and functions of hemicelluloses at the molecular level make wood mechanically strong and tough, which can serve as an ideal building block to design functional materials.

**Figure 1:**
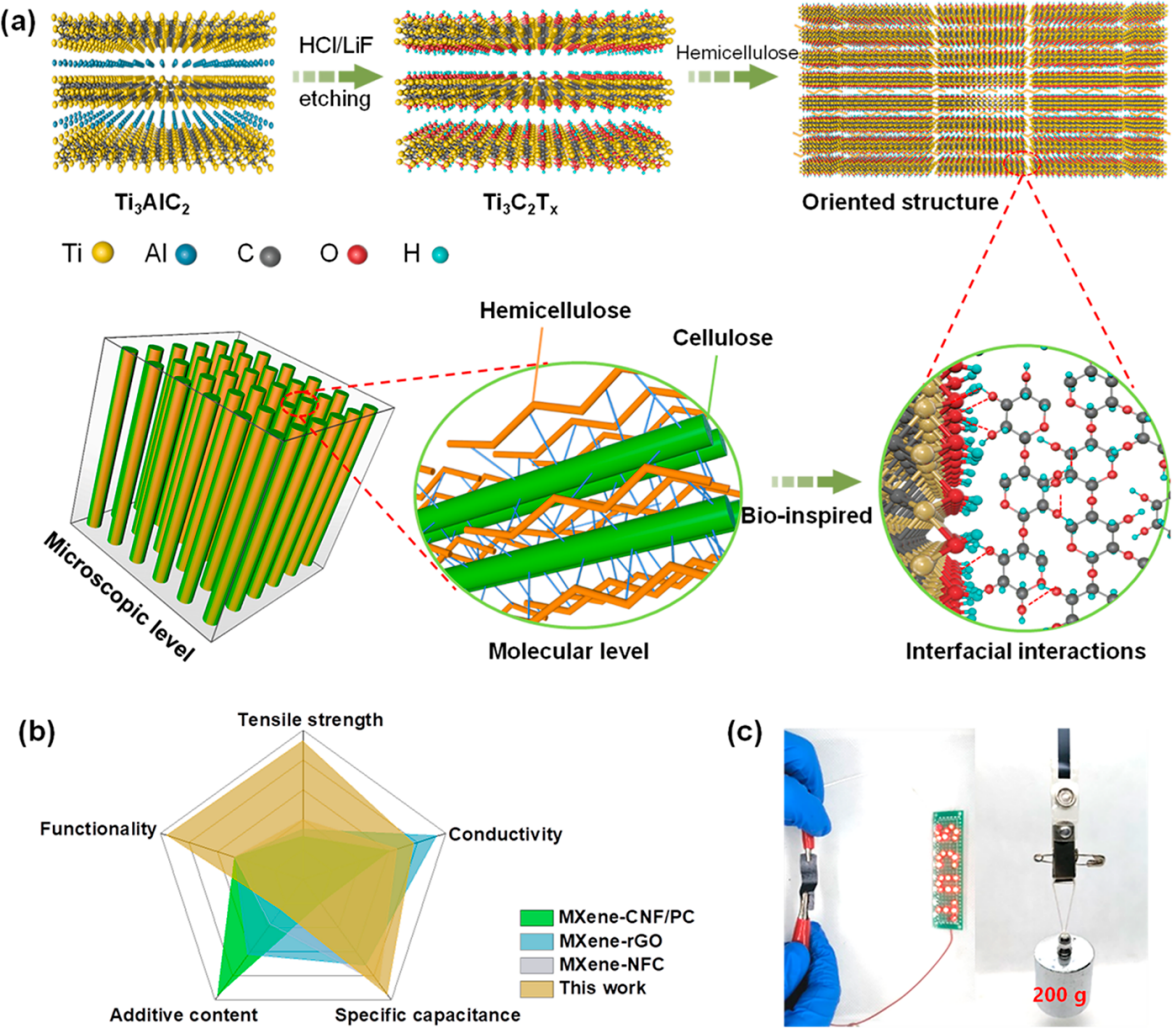
Design of MXene–hemi composite films. (a) Schematic illustration of wood inspired MXene–hemi composite films with oriented structures at the microscopic level and interfacial interactions at the molecular level. (b) Radar chart to compare five parameters of as-designed MXene–hemi films and other reported MXene-based composite films. (c) Photograph of the MXene–hemi films lighting a LED logo and with a loading weight of 200 g.

Inspired by wood, Ti_3_C_2_T_*x*_ MXene–hemicellulose (MXene–hemi) composite films are obtained via a simple vacuum-assisted self-assembly process. Hemicellulose (*xylo*-oligosaccharide) is a mixture of oligomers composed of xylose units connected by β-1,4 glycosidic bonds, normally including 2–7 branched or linear monosaccharide units (Figures S1–S3 and Tables S1 and S2). After self-assembly, hemicellulose with abundant oxygen-containing functional groups is embedded in oriented MXene nanosheets, tethering together adjacent nanosheets through hydrogen bonding (Figure 1a). Such an oriented “reinforced concrete” structure with strong interfacial interactions significantly enhances the mechanical strength. In addition, compared with poly(vinyl alcohol) and other macromolecules, water-soluble hemicellulose is short-chained with a low degree of polymerization. This offers the possibility of tethering MXene nanosheets into robust structural materials without introducing a large number of insulating phases among adjacent MXene sheets and thus shows relatively weakening effect in conductivity (Table S3). Interestingly, the intercalation of hygroscopic hemicellulose among conductive MXene layers provides more moisture adsorption sites and promotes enlarging the interlayer spacing, endowing the composite film with a fast humidity response performance. As a result, the flexible MXene–hemi film can simultaneously integrate a high mechanical strength (125 MPa), high electrical conductivity (64,300 S m^–1^), high gravimetric capacitance (335 F g^–1^), and multifunctionality when serving as a structural electrode with a very low hemicellulose content (Figure 1b-c).^26,27^

## Results and Discussion

**Figure 2:**
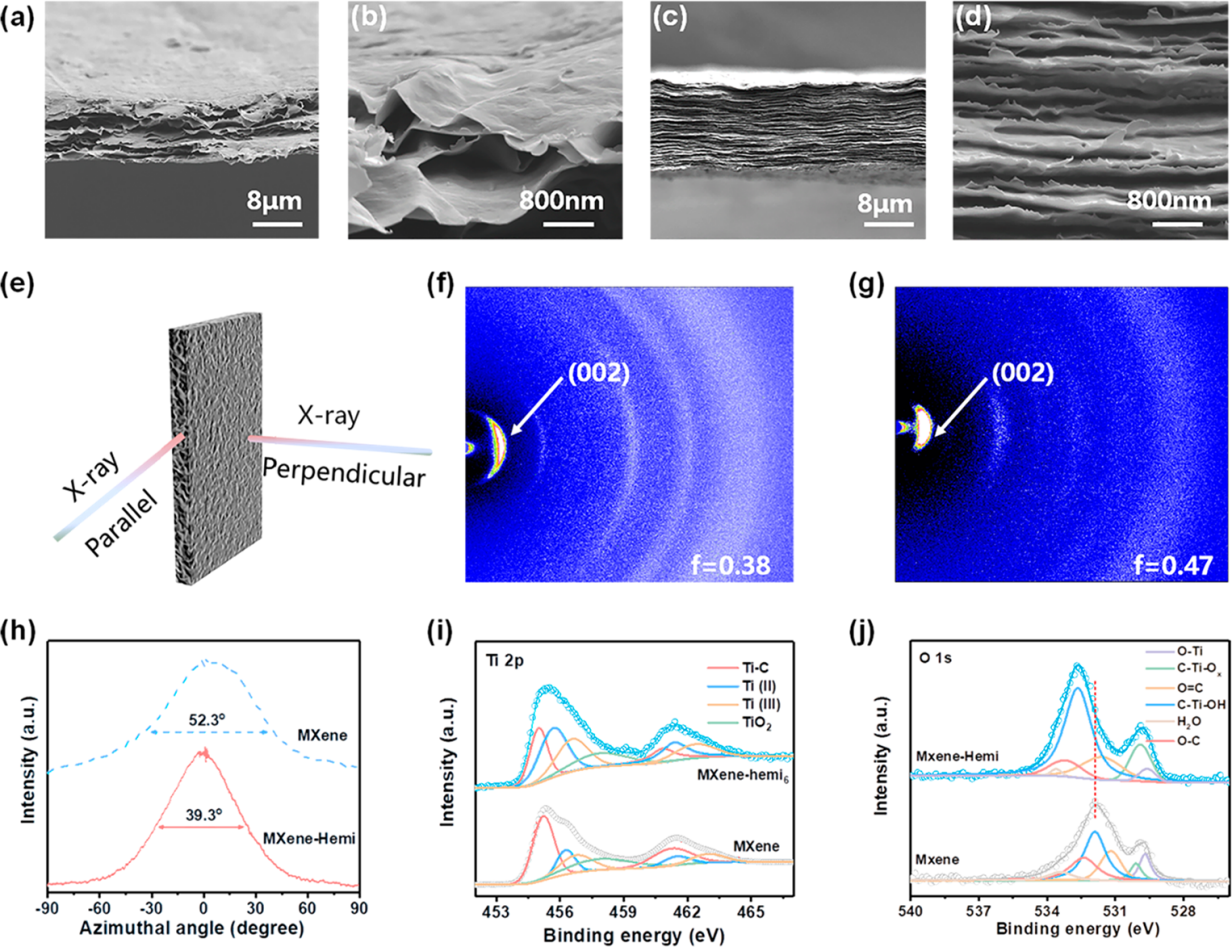
Characterization of MXene–hemi and MXene films. (a,b) Cross-sectional SEM images of different MXene films: (a,b) pristine MXene films, (c,d) MXene–hemi films. (e) Schematic illustration of the position of the X-ray of wide-angle X-ray diffraction (WAXD). WAXD patterns of different MXene films when the X-ray is parallel to the film’s plane: (f) pristine MXene films, (g) MXene–hemi films. (h) Azimuthal plots for the (002) peaks of pristine MXene and MXene–hemi films. (i,j) XPS spectra of MXene–hemi and pristine MXene films.

Ti_3_C_2_T_*x*_ MXene was prepared by selectively etching Al layers from the MAX phase (Ti_3_AlC_2_) via the HCl/LiF solution (Figure 1a).^28^ From the XRD patterns, the negligible (104) peak located at 38.8° and the obvious shift of (002) from 9.5° to 8.5° after etching (Figure S4) indicate the effective etching Al layers in the MAX phase, which is consistent with previous works.^29−32^ After subsequent sonication, the obtained Ti_3_C_2_T_*x*_ show nearly transparent platelike morphology with lateral sizes between several hundreds of nanometers and several micrometers (Figure S5a–c). The morphology and dimension of Ti_3_C_2_T_*x*_ nanosheets are also proved by the AFM image (Figure S5d). After the synthesis of Ti_3_C_2_T_*x*_ nanosheets, MXene films are fabricated by a vacuum-assisted filtration process. Pristine MXene film shows a disordered and loosely lamellar structure with a rich interlayer space (Figure 2a,b). In a sharp contrast, MXene–hemi films gradually display an oriented and densely packed lamellar structure without an obvious gap, which highlights the significance of hemicellulose (Figure 2c,d and Figure S6). Furthermore, we employed wide-angle X-ray diffraction (WAXD) characterization to evaluate the oritention more quantitatively.^33^ Specifically, the characterization was carried out at two directions (Figure 2e). Compared with the perpendicular configuration, both films exhibit strong diffraction intensities and arcs when the X-ray is parallel to the film plane (Figure S7). Notably, the MXene–hemi films shows a narrower arc and full width at half-maximum (39.3°) compared to pristine MXene films, which indicates that MXene nanosheets in MXene–hemi films are more preferentially oriented along the in-plane direction (Figure 2f–h). To further study the orientation of the MXene films, Herman’s orientation factor (*f*) was extracted from the azimuthal plots of the (002) peak (Figure 2h).^34^ The orientation factor of MXene–hemi films (0.47) was slightly higher than that of pristine MXene films (0.38), indicating an enhancement of the orientation of the microscopic structure after the incorporation of hemicellulose.^35^

The origin of the enhancement of the orientation is evaluated by X-ray photoelectron spectroscopy (XPS). The signals for C, O, F, and Ti can be detected in all samples, revealing the successful generation of abundant polar groups (−OH, −F, −O) on the MXene nanosheets during the etching process (Figure S8a).^36^ These polar groups play a vital role in interacting with hemicellulose. The deconvolution of Ti 2p shows mixed moieties of Ti–C, Ti(II), Ti(III), TiO_2_, and Ti–C species (Figure 2i).^37^ Compared with pristine MXene films, the components of Ti(II) and Ti(III) are gradually increased (from 33% to 59%), while that of Ti–C is decreased (from 44% to 24%, Figure S8b–d). These results demonstrate that the local chemical environments for Ti–C of MXene have been changed for MXene–hemi films, forming more interactions with polar groups (−O, −OH). In the case of O 1s spectra, a sharp increase of the C–Ti–OH peak is consistent with Ti 2p (Figure 2j).^38^ In addition, upshifts toward higher binding energies were monitored for the O 1s peak, further proving the formation of hydrogen-bonding connections between MXene and hemicellulose. Therefore, hemicellulose serving as a molecular binder can increase interfacial interactions and tightly bind adjacent MXene nanosheets together, leading to an enhancement of the orientation and the reduction of the interlayer gaps.

**Figure 3:**
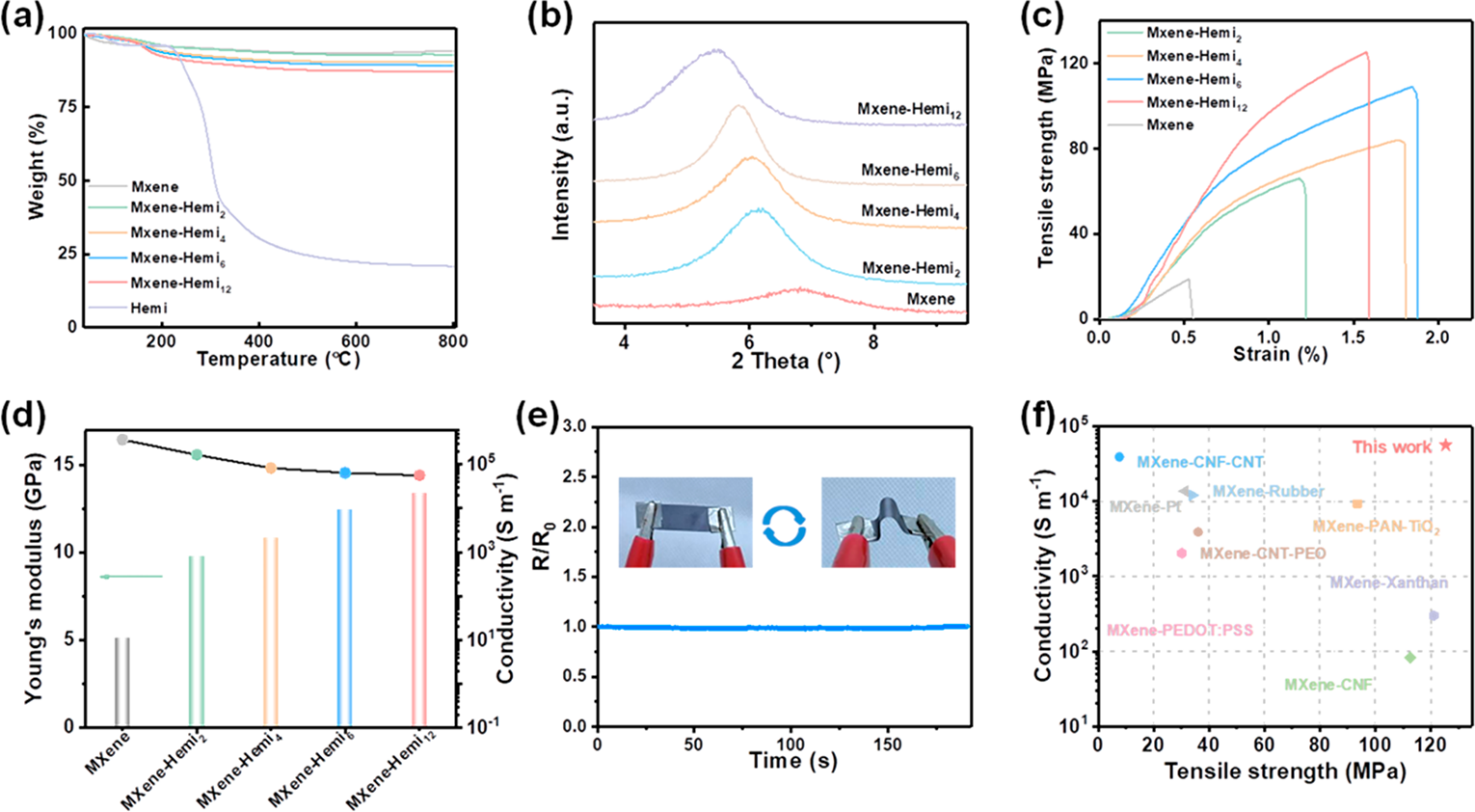
Mechanical properties and electrical conductivity. (a–c) TGA curves, XRD patterns, and tensile stress–strain plots of MXene films with different amounts of hemicellulose. (d) Comparison of Young’s modulus and electrical conductivity of MXene films with different amounts of hemicellulose. (e) Real-time relative resistance of the MXene–hemi film upon repeated bending. (f) Comparison of the electrical conductivity versus tensile strength of the MXene–hemi film with other reported MXene-based films.

After verifying the oriented microstructures and molecular interactions, the electrical conductivity and mechanical properties of MXene films were further studied. Figure 3a shows the thermogravimetric analysis (TGA) curves of MXene films with different amounts of hemicellulose. Compared with MXene, hemicellulose suffers from significant weight loss in the range of 200–800 °C. According to the calculations, the weight percent of hemicellulose of MXene–hemi_2_, MXene–hemi_4_, MXene–hemi_6_, and MXene–hemi_12_ corresponds to 1.5%, 5%, 7%, and 10%, respectively. As shown in XRD patterns, pristine MXene film displays a peak at 6.8° (*d*-spacing of 13.0 Å) related to (002) planes (Figure 3b).^36^ This peak shifted from 6.2° (*d*-spacing of 14.3 Å) to 5.5° (*d*-spacing of 16.1 Å) when the concentration of hemicellulose increased from 2 to 12 mg mL^–1^ (Figure 3b and Table S4). These results present a large amount of hemicellulose molecules embedded into adjacent MXene nanosheets as a function of hemicellulose content, which increases the interlayer spacing.

The incorporation of hemicellulose into MXene films significantly enhanced their mechanical properties (Figure 3e). From Figure 3c, the tensile strength increases from 66 MPa for MXene–hemi_2_ to 125 MPa for MXene–hemi_12_, which is 6.6 times higher than that of the original MXene films (19 MPa). Similarly, Young’s modulus of MXene films also increases with increasing hemicellulose content (Figure 3d). The outstanding mechanical properties are ascribed to highly oriented microstructures and improved molecular interactions. More importantly, MXene–hemi films inherit the inherent high electrical conductivity of MXene. Pristine MXene film has a conductivity of 361,218 S m^–1^, a 2 mg mL^–1^ hemicellulose concentration slightly decreases the electrical conductivity to 180,676 S m^–1^, and after increasing the hemicellulose concentration to 12 mg mL^–1^, a high electrical conductivity of 64,300 S m^–1^ can be still retained (Figure 3d). Unlike other macromolecules, increased mechanical strength usually compromises the electrical conductivities by several orders of magnitude. For example, the electrical conductivity of polyacrylonitrile@polydopamine intercalated MXene film dropped to 9,268 S m^–1^ when the strength increased to 93.55 MPa.^39^ Likewise, the conductivity of the nanocellulose intercalated MXene film dropped sharply to 621 S m^–1^ when the strength improved to 112.5 MPa.^40^ The water-soluble hemicellulose is short-chained with a low degree of polymerization. This offers the possibility of tethering MXene nanosheets into robust materials without introducing a large number of insulating phases. Therefore, as-developed MXene–hemi film exhibits synergistically high mechanical properties and high electrical conductivity when compared to other reported MXene-based films, which is a prerequisite for the structural electrodes (Figure 3f).^39−46^

**Figure 4:**
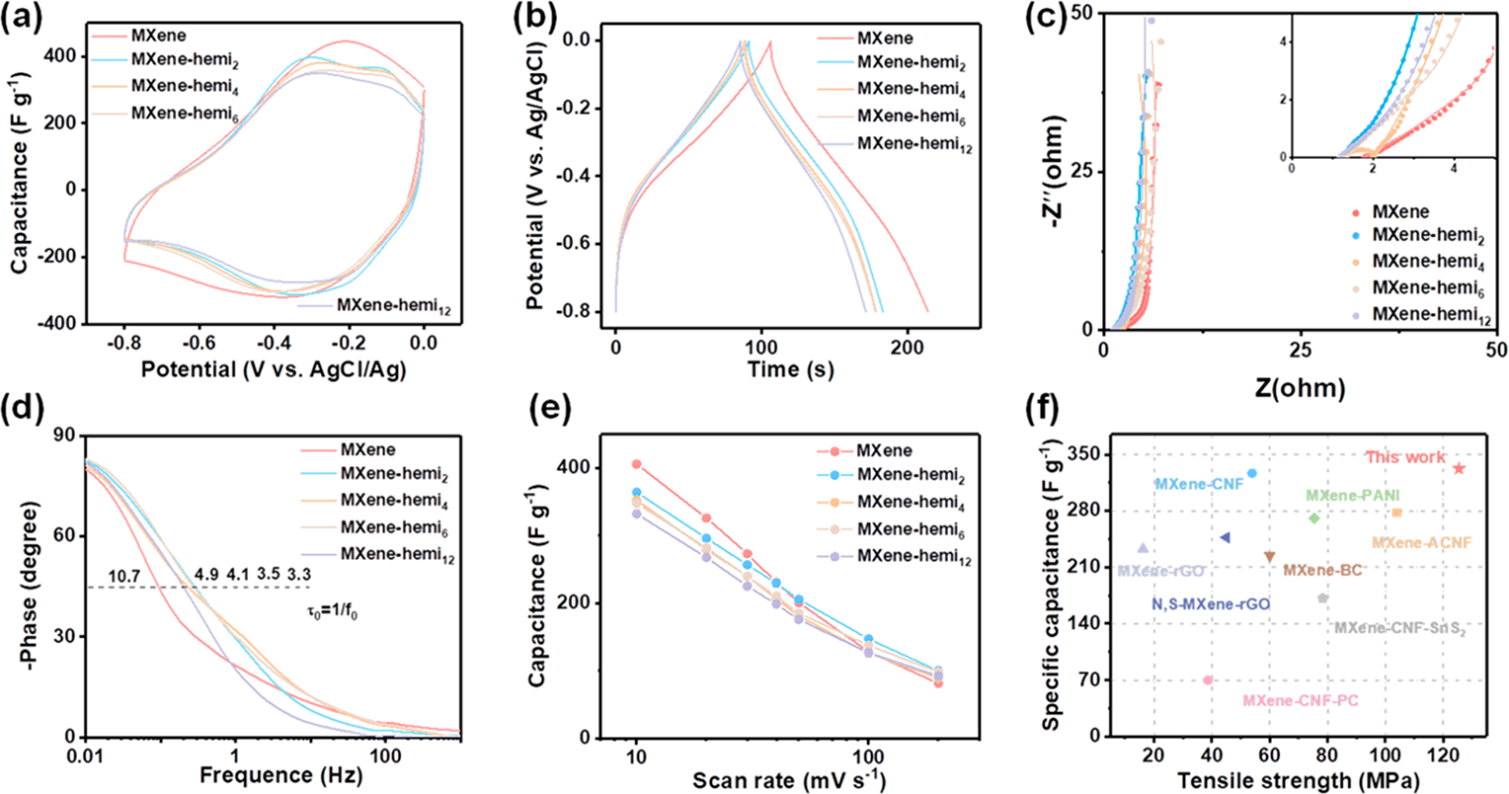
Electrochemical performances of MXene–hemi film electrodes. (a) Cyclic voltammogram (CV) curves at the scan rate of 10 mV s^–1^. (b) Galvanostatic charge/discharge (GCD) curves at the current density of 2 A g^–1^. (c) Electrochemical impedance spectroscopy (EIS). (d) Bode plots. (e) Gravimetric capacitance. (f) Comparison of the capacitance versus tensile strength of the MXene–hemi film with MXene-based films in the literature.

To explore their potential as structural electrodes, the electrochemical properties of MXene–hemi films were evaluated in a three-electrode configuration by using 1 M H_2_SO_4_ as the electrolyte. Figure 4a shows the CV curves of MXene-based films. The incorporation of hemicellulose does not dramatically deteriorate the specific capacitance of MXene–hemi films compared to the pristine MXene film. Similar results can also be observed from the GCD curves (Figure 4b). Figure 4c displays the EIS of different electrodes. Both electrodes have a small internal resistance (*R*_*s*_), which is consistent with the high conductivity of MXene film and MXene–hemi films.^47,48^ As for MXene–hemi electrodes, notably, the 45° angle section at the midfrequency ranges significantly reduces. In addition, the slope of the oblique line at low frequencies slightly increases when compared to the pristine MXene electrode, indicating a lower ion transport resistance (Figure S9).^49−51^ In order to gain insights into the ion resistance, a complex capacitance analysis and frequency response were conducted. The electrodes with hemicellulose show higher phase angle values at low frequencies and lower time constant (τ_0_), proving better ion response and kinetics (Figure 4d).^52^ To further quantitatively calculate the ionic resistance (*R*_*i*_), the dependence of the imaginary capacitance on the real impedance was correlated (Figure S10).^53^ Among them, electrodes with hemicellulose exhibit lower *R*_*i*_ values, further proving lower ion transport resistance. Hydrophilic hemicellulose can act as a molecular spacer among MXene nanosheets with increased interlayer spacing, which may facilitate ion transport. As a result, MXene–hemi electrodes possess a better rate performance at high scan rates (Figure 4e and Figure S11). Moreover, gravimetric capacitances were measured of 366, 355, and 350 F g^–1^ for MXene–hemi_2_, MXene–hemi_4_, and MXene–hemi_6_ electrodes, respectively. A high capacitance of 335 F g^–1^ was retained for the MXene–hemi_12_ electrode when compared to that of the pristine MXene electrode with only 17% lower gravimetric capacitance (405 F g^–1^). However, the mechanical strength of the MXene–hemi_12_ electrode increased by 660% from 19 to 125 MPa. Therefore, the MXene–hemi electrode also shows decent integration of the high specific capacitance and mechanical strength, which is better than that for other reported MXene-based composite electrodes (Figure 4f).^26,27,54−59^

**Figure 5:**
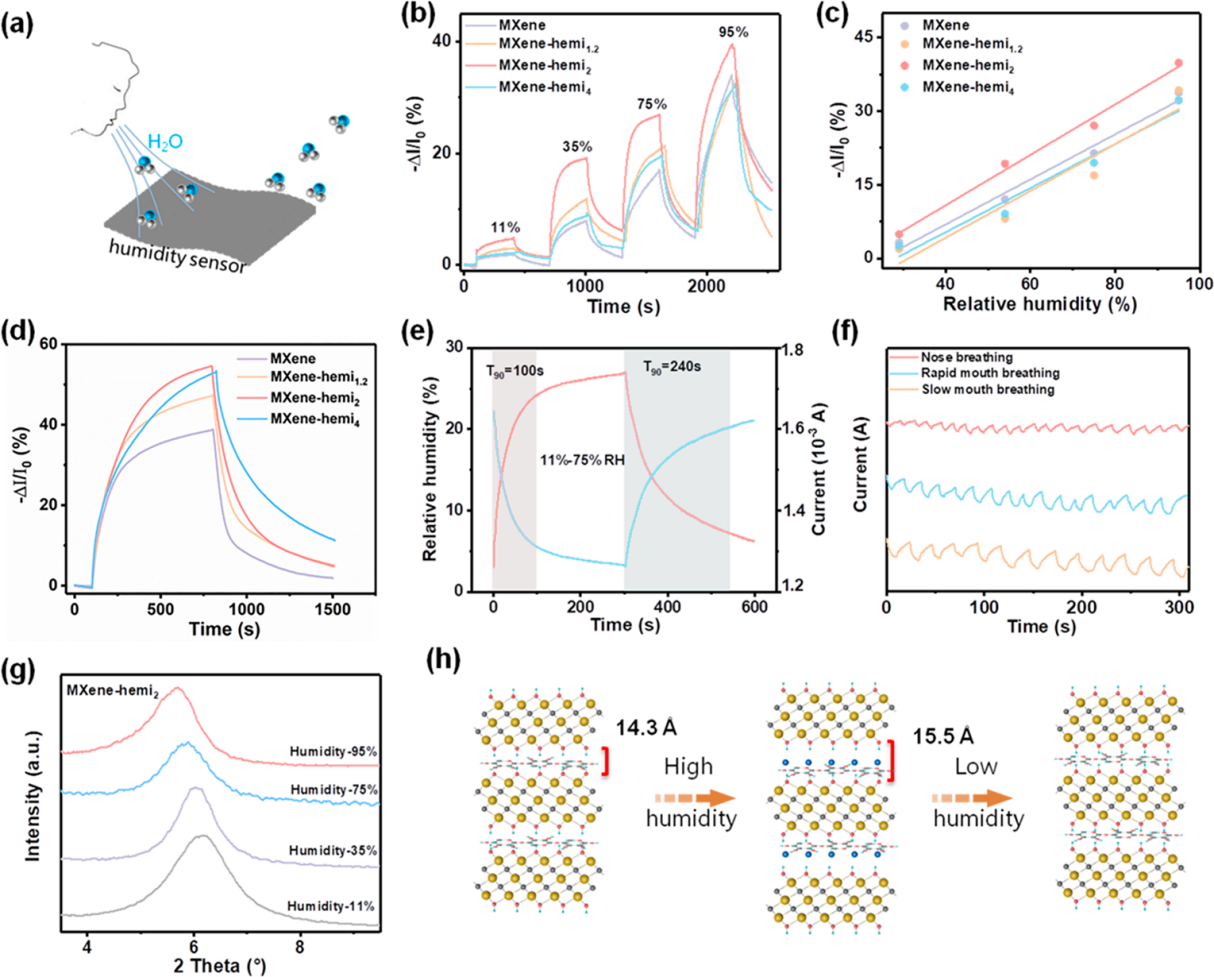
Humidity sensitive response of the MXene–hemi films. (a) Schematic illustration of MXene–hemi film-based sensor. (b) Dynamic response and recovery performance of MXene–hemi films with 11%, 35%, 75%, and 95% relative humidity. (c) Response of MXene–hemi films as a function of relative humidity. (d) Response and recovery performance of MXene–hemi films between 11% and 95% relative humidity. (e) Response and recovery performance of the MXene–hemi_2_ film between 11% and 75% relative humidity. (f) Current–time response curves of the MXene–hemi_2_ film under human breathing. (g) XRD patterns of the MXene–hemi_2_ film under different relative humidity values. (h) Schematic illustrations of the response mechanism of MXene–hemi films.

In addition to imparting excellent mechanical properties to the composite film, hemicellulose also exhibits rapid moisture absorption, which in turn brings fast humidity-responsive properties to the composite film (Figure S12). The humidity-response property of the MXene–hemi films was investigated through monitoring their electrical signal changes under various relative humidity values (Figure 5a).^60^Figure 5b shows the dynamic response curves of different films under various relative humidity values of 11%, 35%, 75%, and 95%. All the films are sensitive to the variations in relative humidity and achieve the largest response value at 95% relative humidity. Moreover, the response values increase linearly with relative humidity, suggesting outstanding sensitivity (Figure 5c).^61^ Compared with pristine MXene film, all the films with hemicellulose show a greater response. In particular, MXene–hemi_2_ film shows clear plateaus and the highest response values under identical conditions (Figureb 5b,d). Comprehensively, MXene–hemi_2_ film shows the largest sensitivity and fastest response.

Besides the responsive property, the response and recovery times that defined the duration of the sensor to achieve 90% of the total response during desorption and absorption processes are also two important parameters to illustrate the sensing performance.^62^ From Figure 5e, the response and recovery times are calculated to be 100 and 240 s, respectively. These response–recovery times are comparable to other sensors.^63−65^ To explore the responsive property of the sensor at real working circumstances, MXene–hemi_2_ film was demonstrated to detect mouth and nose breathing. As can be seen, the breathing types and breathing rates can be clearly distinguished from the curves (Figure 5f). The underlying response mechanism was further investigated by using XRD patterns. The XRD pattern of the MXene–hemi_2_ film at low relative humidity (11%) shows a peak at 6.2° (*d*-spacing of 14.3 Å, Figure 5g). This peak shifted from 6.0° (*d*-spacing of 14.7 Å) to 5.7° (*d*-spacing of 15.5 Å) when the relative humidity increased from 35% to 95% (Figure 5g and Table S5). These results reveal that a large amount of water molecules were uptaken into the adjacent MXene nanosheets as a function of relative humidity, resulting in an increase in the interlayer spacing and resistance of the film (Figure 5h). Compared with pristine MXene films, the intercalation of hygroscopic hemicellulose increases the interlayer spacing of the composite films while enhancing the capture ability of water molecules. Nevertheless, more hemicellulose content can capture more water molecules but correspondingly prolong the response time, and thus, the MXene–hemi_2_ film exhibits the highest sensitivity and fastest response.

## Conclusions

In summary, inspired by wood structures from the molecular to the macroscopic levels, we have demonstrated a hemicellulose-intercalated MXene film with synergistically high mechanical properties and high electrical conductivity as an electrode for structural supercapacitor and humidity sensor. The significantly enhanced mechanical properties are attributed to the improved orientation at the microscopic level and the increased interfacial interactions at the molecular level. In addition, different from other macromolecular polymers, hemicellulose is short-chained with a low degree of polymerization, offering the possibility of tethering MXene nanosheets into robust materials without introducing a large number of insulating phases. Moreover, by taking advantage of the hygroscopic hemicellulose, the composite film also showed excellent humidity-responsive properties. This work provides an alternative strategy for the fabrication of robust MXene-based structural materials for multifunctional electronic applications.

## Methods

### Synthesis of Ti_3_C_2_T_*x*_ MXene

Ti_3_AlC_2_ powder (300 mesh) was obtained from Laizhou Kai Kai Ceramic Materials Co., Ltd. The Ti_3_C_2_T_*x*_ MXene dispersion in water solution was prepared according to a previous work.^66^ For detailed synthesis procedures, see the Surpporting Information.

### Preparation of MXene–Hemicellulose Films

Hemicellulose (xylo-oligosaccharide), obtained from corncob through pretreatment, enzymatic hydrolysis, flocculation, and subsequent purification, was purchased from Shanghai Yuanye Bio-Technology Co. MXene–hemicellulose films were prepared by adding different amounts of hemicellulose (*xylo*-oligosaccharide) into the same volume of MXene dispersion (2 mg/mL, 20 mL). The concentration of hemicellulose was controlled at 2, 4, 6, and 12 mg/mL, respectively. The composite films were obtained through simple vacuum filtration on a hydrophobic polyvinyl difluoride (PVDF) membrane with a pore size of 0.22 μm. The films were dried in a vacuum system and peeled off from the PVDF membrane. The obtained films were named MXene–hemi_2_, MXene–hemi_4_, MXene–hemi_6_, and MXene–hemi_12_, respectively. For comparison, pristine MXene film was prepared without hemicellulose under the same conditions.

## Supporting Information Available

Characterization, mechanical, and electrochemical properties of hemicellulose the exfoliated MXene and MXene-based films (Figures S1–S12, Tables S1–S5) (PDF)
