# Supplementary material for: Robust Bioinspired MXene–Hemicellulose Composite Films with Excellent Electrical Conductivity for Multifunctional Electrode Applications
Source: ACS Nano. 2022 Oct 26;16(11):19124–32. doi: 10.1021/acsnano.2c08163 (PMC9706662; doi:10.1021/acsnano.2c08163)
Supplement: Supplementary file 1 — nn2c08163_si_001.pdf [file nn2c08163_si_001.pdf]

Supporting Information

Robust bioinspired MXene-hemicellulose composite films with excellent electrical conductivity for multifunctional electrode applications

Ruwei Chen, Hao Tang, Yuhang Dai, Wei Zong, Wei Zhang, Guanjie He, and Xiaohui Wang**

Methods

Synthesis of $Ti_3C_2T_x$ MXene. Briefly, 3.2 g of LiF powder was added into 40 ml of 9 M HCl solution. Then, the mixture was stirred for several minutes until LiF was fully dissolved. The mixture was transfered into an ice bath and 2 g of Ti_3AlC_2 MAX powder (~300 mesh) was added within 20 minutes. Then, the mixed solution was stirred for 24 h at 35 ℃. Afterwards, the mixture was washed with DI water until the pH of the supernatant to reach ~6. Subsequently, the washed precipitate was added to 100 ml DI water and tip-sonicated in an ice bath for 1h. At last, the sonicated mixture was centrifuged at 3,500 rpm for 1 h and the supernatant was collected for further use.

Fabrication and measurement of humidity sensors. MXene-based films were cut into rectangular sheets (20×5 mm^2). The conductive silver paste was painted on both ends of the films to connect two copper wires. The obtained sensors were dried overnight at 40 ℃ in a vacuum oven before use.

The different humidity conditions were controlled by saturated solutions of different salts: LiCl (11%), $MgCl_2$ (35%), NaCl (75%) and KNO_3 (95%). The testing sensors were put into the sealed glass bottle containing corresponding saturated salt solutions, and two ends

were connected with a potentiostat. The responses were recorded by changing the current flow through the sensors under an applied voltage of 0.01V. The response was calculated according to the following equation:

$$\text{Response} = \frac{-\Delta \text{I}}{\text{I}_0}$$

Where $\Delta \text{I} = \text{I}_{\text{RH}} - \text{I}_0$, I_0 is the initial current of the samples at 11% relative humidity. I_{RH} is the current upon exposure to the targeted relative humidity conditions.

Characterizations

Conductivity measurements. The sheet resistance of MXene-based films was tested by a four-probe method (RTS-8, 4 PROBE TECH, China). Electronic conductivity σ (S/m) was calculated according to the following equations:

$$\sigma = \frac{1}{\text{R}_\text{s} * \text{d}}$$

where Rs (Ω/sq) is the measured sheet resistance, and d is the thickness of MXene-based films.

X-ray diffraction measurement. X-ray powder diffraction was conducted by an X-ray diffractometer (Bruker D8 Advance). The interlayer d-spacing (Å) of MXene-based films was calculated according to the following equation:

$$\text{d} = \frac{\lambda}{\sin 2\theta}$$

where λ (1.5406 Å) is the wavelength of X-ray, and 2θ is the scattering angles of the (0002) peak.

Two-dimensional wide-angle X-ray diffraction (2D WAXD, HomeLab, Rigaku Denki, Japan) was carried out to evaluate the orientation of MXene-based film. The 2D scattering images were analyzed with Fit2D software from the European Synchronization Radiation Facility. The Herman's orientation parameter (f) from the azimuthal-integrated intensity distribution curves were calculated by using the following equation:

$$f = \frac{3\cos^2\varphi - 1}{2}$$

$$\cos^2\varphi = \frac{\sum_0^{\pi/2} I(\varphi)\sin\varphi\cos^2\varphi}{\sum_0^{\pi/2} I(\varphi)\sin\varphi}$$

Where φ is the azimuthal angle, and I(φ) is the intensity distribution along with φ.

Electrochemical measurements. Electrochemical workstation (CHI 660E) was used for all electrochemical measurements. Three-electrode tests were performed in plastic Swagelok cell where glassy carbon electrodes were used as current collectors, MXene-based films were punched to desired size and directly used as the working electrode. A overcapacitive activated carbon electrode and Ag/AgCl were used as the counter electrode and reference electrode, respectively. The specific capacitances were calculated from the CV curves according to the following equation:

$$C = \frac{1}{\Delta v}\int \frac{jdV}{s}$$

Where C is the specific capacitance (F/g), ΔV is the voltage window (V), j is the corresponding current density (A/g), V is the voltage, and s is the scan rate (V/s).

Other characterizations. The microstructures were investigated using scanning electron microscopy (SEM, ZEISS Merlin, Oberkochen, Germany) and transmission electron microscopy (TEM, JEM-2100, JEOJ Ltd., Tokyo, Japan). X-ray photoelectron spectroscopy (XPS) was conducted on an AMICUS (Shimadzu, Japan) spectrometer with monochromated Mg Kα radiation. The tensile strength of the aerogel was measured using a tensile compression material testing machine (Instron Universal Testing Machine 5565, USA).

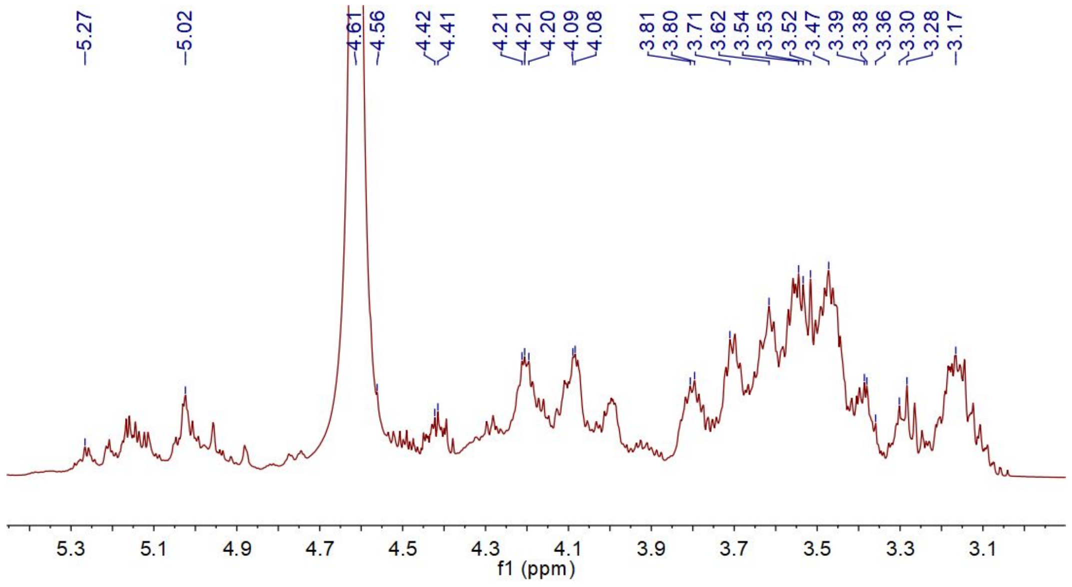

Figure S1. ^{1}H-NMR spectrum of hemicellulose (xylo-oligosaccharide).

Table S1. Detailed attribution of ^{1}H-NMR spectrum peaks of hemicellulose (xylo-oligosaccharide).

Attribution of sugar group		chemical shift (ppm)
Reducing terminal group of xylose X-α	H-1	5.02
Reducing terminal group of xylose X-β	H-1	4.56
Non reducing terminal group of xylose	H-1	4.41-4.42
	H-1	5.27
	H-2	4.08
Arabinose group	H-3	3.81
	H-4	4.21
	H-5	3.80
	$-OCH_3$	3.38
	H-2	3.14
β- (1-4) - D-xylosyl	H-3	3.47
	H-4	3.71
	H-5	3.30
	H-3	3.36
β- D-xylose non reducing terminal group	H-4	3.53
	H-5	3.28

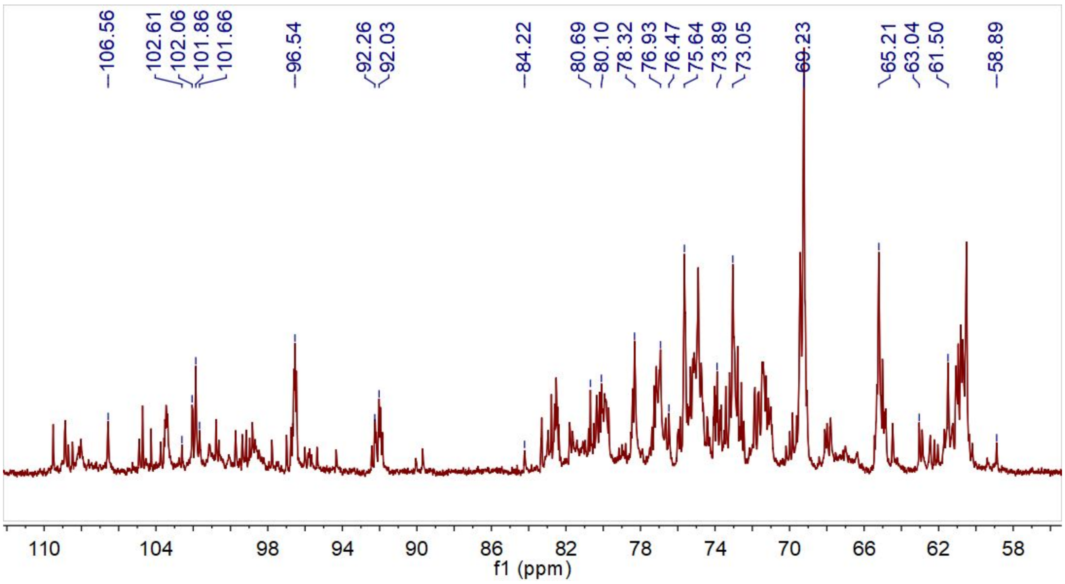

Figure S2. ^{13}C-NMR spectrum of hemicellulose (xylo-oligosaccharide).

Table S2. Detailed attribution of ^{13}C-NMR spectrum peaks of hemicellulose (xylo-oligosaccharide).

Attribution of sugar group		chemical shift (ppm)
Reducing terminal group of xylose X-α	C-1	92.26
Reducing terminal group of xylose X-β	C-1	96.54
Non reducing terminal group of xylose	C-1	101.66-102.61
	C-1	106.56
	C-2	80.69
Arabinose group	C-3	78.32
	C-4	84.22
	C-5	61.50
	$-OCH_3$	58.89
	C-2	73.05
β- (1-4) - D-xylosyl	C-3	73.89
	C-4	76.93
	C-5	63.04
	C-3	75.64
β- D-xylose non reducing terminal group	C-4	69.23
	C-5	65.21

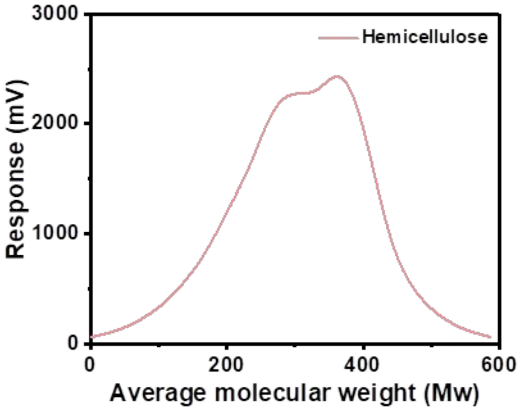

Figure S3. Average molecular weight of hemicellulose (xylo-oligosaccharide).

Table S3. Comparison of mechanical strength and electrical conductivity of our MXene/hemicellulose composite film with other MXene-based films containing different polymer binders.

Materials	Mechanical strength (MPa)	Conductivity (S m^{-1})	Ref
Mxene/CNF/porous carbon	38.6	8310	[1]
Mxene/CNF	53.9	24930	[2]
Mxene/PEDOT:PSS	13.71	34050	[3]
MXene/BN/CNF	27.8	35640	[4]
MXene/polyimide	25	3787.9	[5]
MXene/cellulose/AgNWs	34	58843	[6]
MXene/Methylcellulose	53.2	842	[7]
MXene/CNF	65	24875	[8]
MXene/CNT/CNF	98	2507	[9]
MXene/CNF	112.5	621	[10]
MXene/xanthan	116	11530	[11]
MXene/aramid nanofiber	124	43200	[12]
MXene/CNF	124.6	2600	[13]
MXene/CNF	135.4	739.4	[14]
MXene/hemicellulose	125	64300	This work

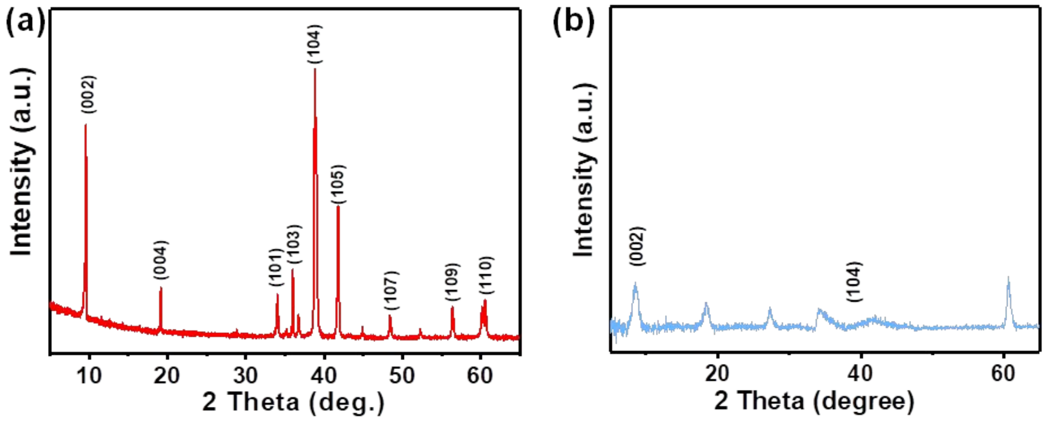

Figure S4. (a) XRD pattern of Ti_3AlC_2 MAX phase. (b) XRD pattern of $Ti_3C_2T_x$ MXene after etching.

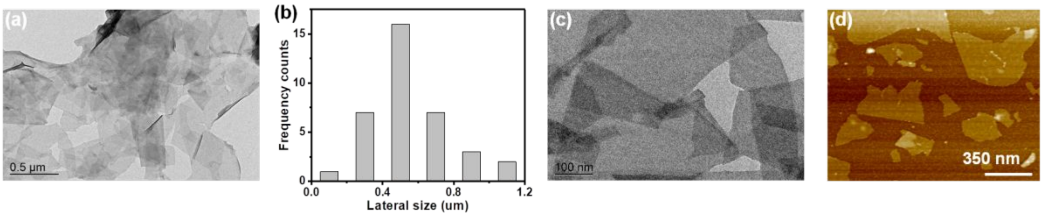

Figure S5. (a) TEM images of $Ti_3C_2T_x$ MXene nanosheets at low magnification. (b) Corresponding lateral size distribution of (a). (c) TEM images of $Ti_3C_2T_x$ MXene nanosheets at high magnification (c) AFM image $Ti_3C_2T_x$ MXene nanosheets.

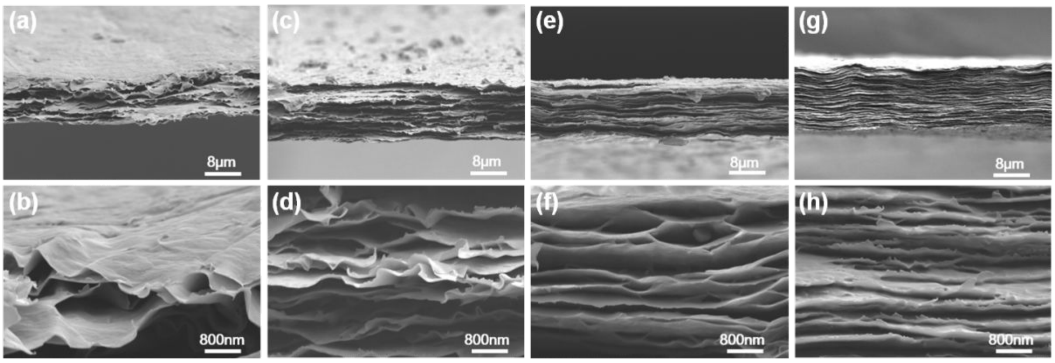

Figure S6. (a-b) SEM images of pristine MXene film. (c-d) SEM images of MXene-hemi$_2$ film. (e-f) SEM images of MXene-hemi$_4$ film. (g-h) SEM images of MXene-hemi$_{12}$ film.

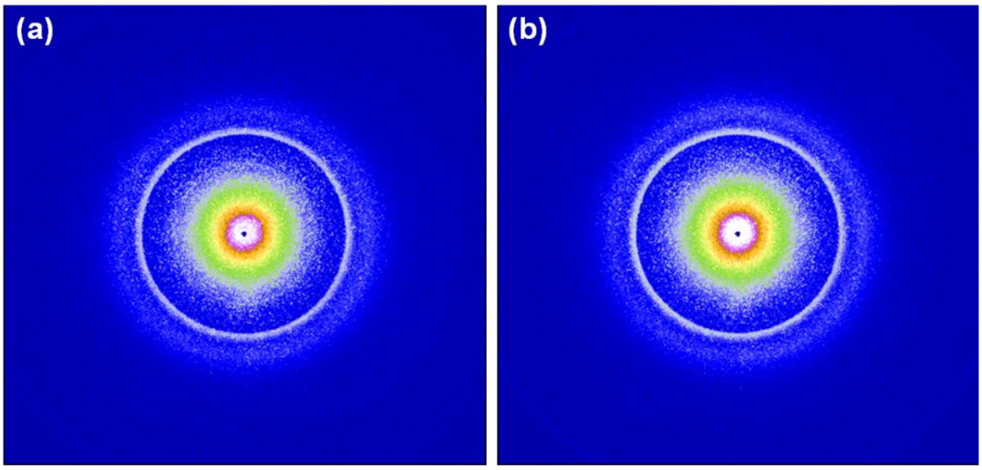

Figure S7. (a) WAXD patterns of pristine MXene film when the film plane is perpendicular to the X-ray beam. (b) WAXD patterns of MXene-hemi$_{12}$ film when the film plane is perpendicular to the X-ray beam.

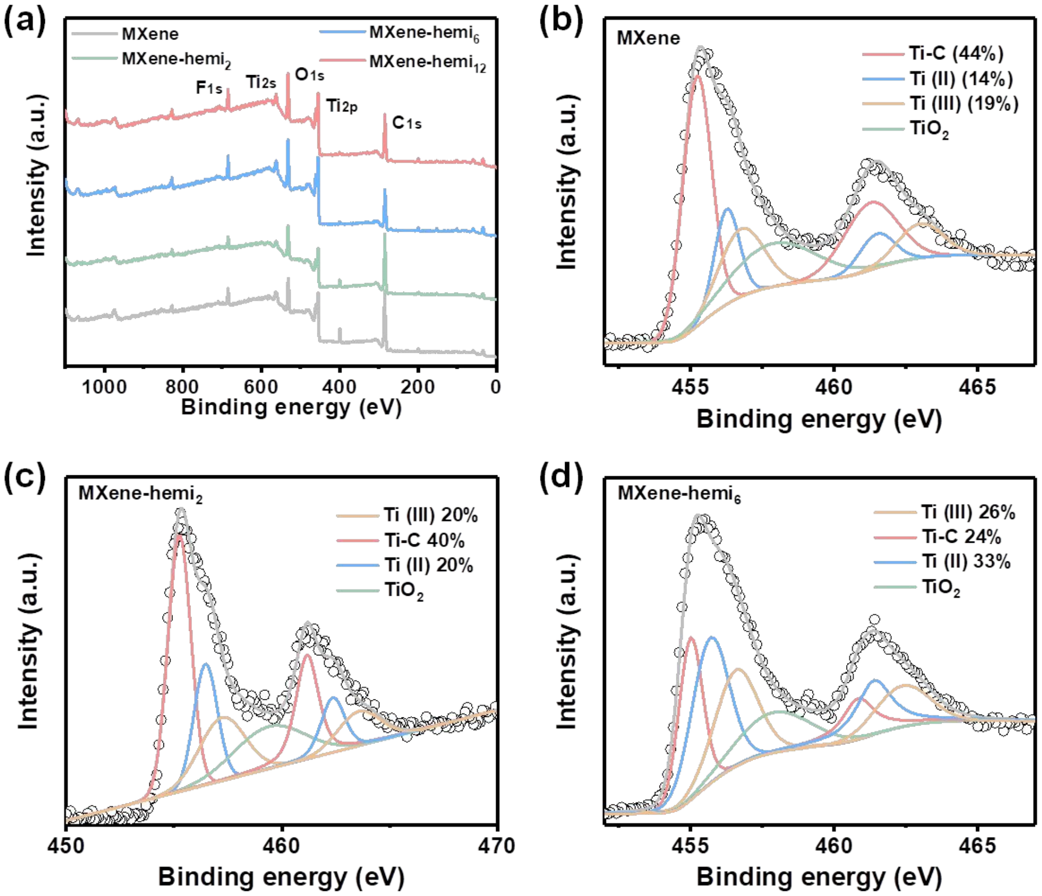

Figure S8. (a) X-ray photoelectron spectroscopy of different films. (b) Ti 2p of pristine MXene film. (c) Ti 2p of MXene-hemi$_2$ film. (d) Ti 2p of MXene-hemi$_6$ film.

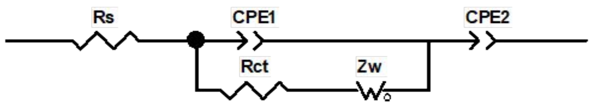

Figure S9. Corresponding equivalent circuit of EIS results.

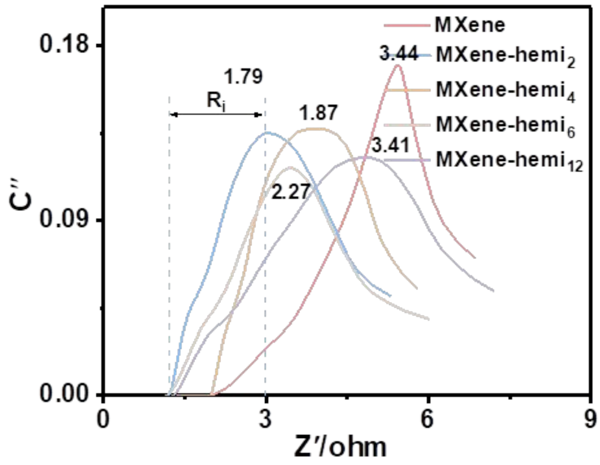

Figure S10. Ionic resistances (Ri): evolution of the imaginary capacitance vs. the real impedance of different samples. The Ri were obtained from the real impedance values between the start of the middle range frequency and f_0 where defines the transition from resistive behavior to capacitive behavior.

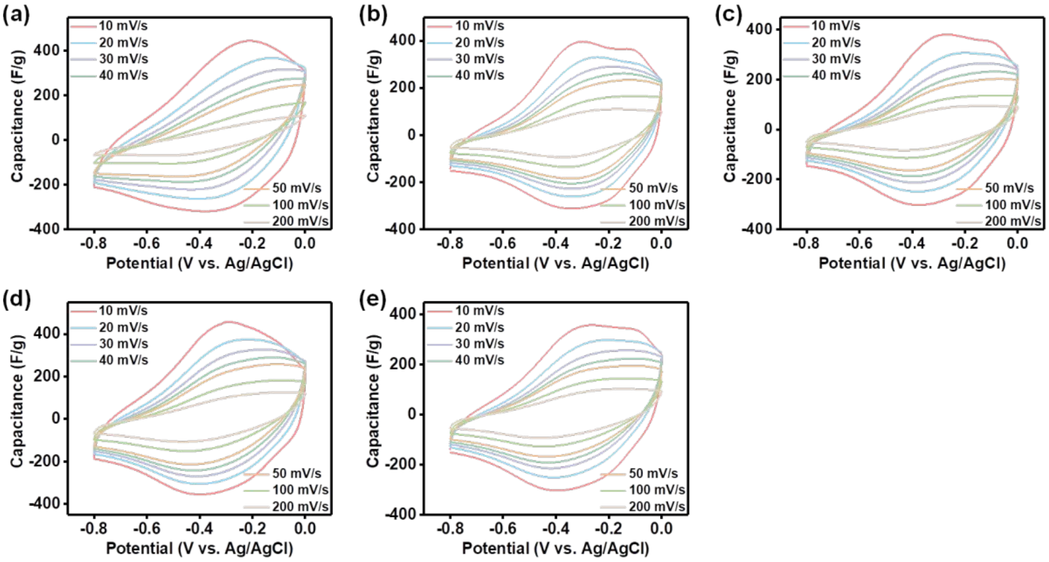

Figure S11. (a) Cyclic voltammogram curves of pristine MXene film at various scan rates. (b) Cyclic voltammogram curves of MXene-hemi$_2$ film at various scan rates. (c) Cyclic voltammogram curves of MXene-hemi$_4$ film at various scan rates. (d) Cyclic voltammogram curves of MXene-hemi$_4$ film at various scan rates. (d) Cyclic voltammogram curves of MXene-hemi$_6$ film at various scan rates. (e) Cyclic voltammogram curves of MXene-hemi$_{12}$ film at various scan rates.

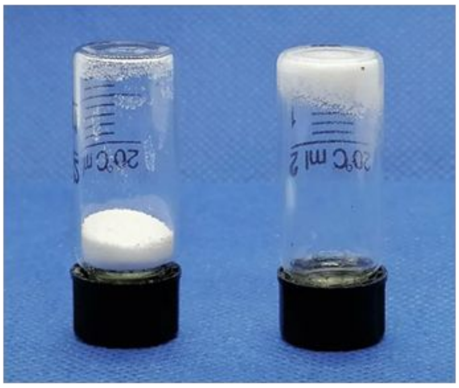

Figure S12. Digital image of hemicellulose before and after moisture absorption.

Table S4. 2θ angle and d-spacing of MXene-based films with different hemicellulose contents.

Samples	2θ (O)	d-spacing (Å)
MXene	6.8	13.0
MXene-hemi$_2$	6.2	14.3
MXene-hemi$_4$	6.0	14.7
MXene-hemi$_6$	5.8	15.2
MXene-hemi$_{12}$	5.5	16.1

Table S5. 2θ angle and d-spacing of MXene-hemi$_2$ film under different relative humidity.

Humidity	2θ (o)	d-spacing (Å)
11%	6.2	14.3
35%	6.0	14.7
75%	5.8	15.2
95%	5.7	15.5

References

(1) Chen, W.; Zhang, D.; Yang, K.; Luo, M.; Yang, P.; Zhou, X. Mxene (Ti_3C_2T)/Cellulose Nanofiber/Porous Carbon Film as Free-Standing Electrode for Ultrathin and Flexible Supercapacitors. *Chem. Eng. J.* **2021**, *413*, 127524.

(2) Chen, J.; Chen, H.; Chen, M.; Zhou, W.; Tian, Q.; Wong, C.-P. Nacre-Inspired Surface-Engineered MXene/Nanocellulose Composite Film for High-Performance Supercapacitors and Zinc-Ion Capacitors. *Chem. Eng. J.* **2022**, *428*, 131380.

(3) Liu, R.; Miao, M.; Li, Y.; Zhang, J.; Cao, S.; Feng, X. Ultrathin Biomimetic Polymeric $Ti_3C_2T_x$ MXene Composite Films for Electromagnetic Interference Shielding. *ACS Appl. Mater. Interfaces.* **2018**, *10* (51), 44787-44795.

(4) Shang, Y.; Ji, Y.; Dong, J.; Yang, G.; Zhang, X.; Su, F.; Feng, Y.; Liu, C. Sandwiched Cellulose Nanofiber/Boron Nitride Nanosheet/$Ti_3C_2T_x$ MXene Composite Film with High Electromagnetic Shielding and Thermal Conductivity yet Insulation Performance. *Compos. Sci.Technol.* **2021**, *214*, 108974.

(5) Sun, K.; Wang, F.; Yang, W.; Liu, H.; Pan, C.; Guo, Z.; Liu, C.; Shen, C. Flexible Conductive Polyimide Fiber/MXene Composite Film for Electromagnetic Interference Shielding and Joule Heating with Excellent Harsh Environment Tolerance. *ACS Appl. Mater. Interfaces.* **2021**, *13* (42), 50368-50380.

(6) Tang, H.; Chen, R.; Huang, Q.; Ge, W.; Zhang, X.; Yang, Y.; Wang, X. Scalable Manufacturing of Leaf-Like MXene/AgNWs/Cellulose Composite Paper Electrode for All-Solid-State Supercapacitor. *EcoMat.* **2022**.

(7) Chen, S.; Ciou, J. H.; Yu, F.; Chen, J.; Lv, J.; Lee, P. S. Molecular-Level Methylcellulose/MXene Hybrids with Greatly Enhanced Electrochemical Actuation. *Adv. Mater.* **2022**, *34* (29), e2200660.

(8) Cui, Z.; Gao, C.; Fan, Z.; Wang, J.; Cheng, Z.; Xie, Z.; Liu, Y.; Wang, Y. Lightweight MXene/Cellulose Nanofiber Composite Film for Electromagnetic Interference Shielding. *J. Electron. Mater.* **2021**, *50* (4), 2101-2110.

(9) Cao, W.; Ma, C.; Tan, S.; Ma, M.; Wan, P.; Chen, F. Ultrathin and Flexible CNTs/MXene/Cellulose Nanofibrils Composite Paper for Electromagnetic Interference Shielding. *Nano-micro. lett.* **2019**, *11* (1), 72.

(10) Zhou, B.; Zhang, Z.; Li, Y.; Han, G.; Feng, Y.; Wang, B.; Zhang, D.; Ma, J.; Liu, C. Flexible, Robust, and Multifunctional Electromagnetic Interference Shielding Film with Alternating Cellulose Nanofiber and MXene Layers. *ACS Appl. Mater. Interfaces.* **2020**, *12* (4), 4895-4905.

(11) Sun, Y.; Ding, R.; Hong, S. Y.; Lee, J.; Seo, Y.-K.; Nam, J.-D.; Suhr, J. MXene-Xanthan Nanocomposite Films with Layered Microstructure for Electromagnetic Interference Shielding and Joule Heating. *Chem. Eng. J.* **2021**, *410*, 128348.

(12) Hu, D.; Wang, S.; Zhang, C.; Yi, P.; Jiang, P.; Huang, X. Ultrathin MXene-Aramid Nanofiber Electromagnetic Interference Shielding Films with Tactile Sensing Ability Withstanding Harsh Temperatures. *Nano Res.* **2021**, *14* (8), 2837-2845.
(13) Liu, D.; Gao, Y.; Song, Y.; Zhu, H.; Zhang, L.; Xie, Y.; Shi, H.; Shi, Z.; Yang, Q.; Xiong, C. Highly Sensitive Multifunctional Electronic Skin Based on Nanocellulose/MXene Composite Films with Good Electromagnetic Shielding Biocompatible Antibacterial Properties. *Biomacromolecules.* **2022**, *23* (1), 182-195.
(14) Cao, W. T.; Chen, F. F.; Zhu, Y. J.; Zhang, Y. G.; Jiang, Y. Y.; Ma, M. G.; Chen, F. Binary Strengthening and Toughening of MXene/Cellulose Nanofiber Composite Paper with Nacre-Inspired Structure and Superior Electromagnetic Interference Shielding Properties. *ACS nano.* **2018**, *12* (5), 4583-4593.
